# Evaluating the coding accuracy of type 2 diabetes mellitus among patients with non-alcoholic fatty liver disease

**DOI:** 10.1186/s12913-024-10634-8

**Published:** 2024-02-16

**Authors:** Seungwon Lee, Abdel Aziz Shaheen, David J. T. Campbell, Christopher Naugler, Jason Jiang, Robin L. Walker, Hude Quan, Joon Lee

**Affiliations:** 1 Department of Community Health Sciences, Cumming School of Medicine, University of Calgary, Calgary, AB T2N 4Z6 Canada; 2 Centre for Health Informatics, Cumming School of Medicine, University of Calgary, Calgary, AB Canada; 3 Alberta Health Services, Calgary, AB Canada; 4 Data Intelligence for Health Lab, Cumming School of Medicine, University of Calgary, Calgary, AB Canada; 5 Department of Medicine, Cumming School of Medicine, University of Calgary, Calgary, AB Canada; 6 Department of Pathology and Laboratory Medicine, Cumming School of Medicine, University of Calgary, Calgary, AB Canada; 7 Department of Cardiac Sciences, Cumming School of Medicine, University of Calgary, Calgary, AB Canada

**Keywords:** Diabetes mellitus, International classification of diseases, Non-alcoholic fatty liver disease, Routinely collected health data, Health services research

## Abstract

**Background:**

Non-alcoholic fatty liver disease (NAFLD) describes a spectrum of chronic fattening of liver that can lead to fibrosis and cirrhosis. Diabetes has been identified as a major comorbidity that contributes to NAFLD progression. Health systems around the world make use of administrative data to conduct population-based prevalence studies. To that end, we sought to assess the accuracy of diabetes International Classification of Diseases (ICD) coding in administrative databases among a cohort of confirmed NAFLD patients in Calgary, Alberta, Canada.

**Methods:**

The Calgary NAFLD Pathway Database was linked to the following databases: Physician Claims, Discharge Abstract Database, National Ambulatory Care Reporting System, Pharmaceutical Information Network database, Laboratory, and Electronic Medical Records. Hemoglobin A1c and diabetes medication details were used to classify diabetes groups into absent, prediabetes, meeting glycemic targets, and not meeting glycemic targets. The performance of ICD codes among these groups was compared to this standard. Within each group, the total numbers of true positives, false positives, false negatives, and true negatives were calculated. Descriptive statistics and bivariate analysis were conducted on identified covariates, including demographics and types of interacted physicians.

**Results:**

A total of 12,012 NAFLD patients were registered through the Calgary NAFLD Pathway Database and 100% were successfully linked to the administrative databases. Overall, diabetes coding showed a sensitivity of 0.81 and a positive predictive value of 0.87. False negative rates in the absent and not meeting glycemic control groups were 4.5% and 6.4%, respectively, whereas the meeting glycemic control group had a 42.2% coding error. Visits to primary and outpatient services were associated with most encounters.

**Conclusion:**

Diabetes ICD coding in administrative databases can accurately detect true diabetic cases. However, patients with diabetes who meets glycemic control targets are less likely to be coded in administrative databases. A detailed understanding of the clinical context will require additional data linkage from primary care settings.

**Supplementary Information:**

The online version contains supplementary material available at 10.1186/s12913-024-10634-8.

## Introduction

Health systems routinely use digital databases to store and code health information. The International Classification of Diseases (ICD) was developed by the World Health Organisation and is used to translate extensive details from electronic medical records (EMR) into standardised codes. ICD codes have been utilized for decades, with ICD code-driven algorithms being routinely employed for identifying chronic conditions, such as the Charlson comorbidity index [1] and the Elixhauser index [2].

Much like healthcare systems worldwide, Canada has multiple administrative health databases that are widely employed in health research. These databases, underpinned by ICD coding, encompass the Discharge Abstract Database (DAD) which contains inpatient data, National Ambulatory Care Reporting System (NACRS) which collects outpatient and emergency department visit details, and Physician Claims which collects details in inpatient and outpatient (e.g., primary care) settings.

ICD codes serve as a common tool for chronic disease and comorbidity surveillance in the populations of both Canada and various other countries [3, 4]. In Canada, national agencies like the Canadian Institute for Health Information have issued directives for coding specifics conditions inclusive of diabetes, leading to the establishment of the National Diabetes Surveillance System [5]. Notably, Type 2 diabetes is strongly associated with non-alcoholic fatty liver disease (NAFLD) [6, 7] and is considered requiring close monitoring for NAFLD [7].

NAFLD, the most common liver disease worldwide [6], is a progressive disease that advances from a non-alcoholic fatty liver to non-alcoholic steatohepatitis (NASH) to NASH with fibrosis [8, 9]. This progression can eventually lead to end stage liver disease or hepatocellular carcinoma [8, 9]. Accurate identification of comorbidity information, such as diabetes, in electronic databases is crucial in this patient population to ensure timely intervention. In Calgary, Canada, a prospective cohort of NAFLD patients from primary care settings has been evaluated for liver fibrosis. Primary Care Providers (PCP) in Calgary are equipped to promptly assess NAFLD patients without a referral to tertiary care [10]. They are also well-informed about the association between diabetes mellitus and NAFLD, and that it is a criterion for NAFLD evaluation (or assessment) [11]. Several studies to date [12, 13] have assessed the accuracy of ICD codes for diabetes diagnoses, but these were related to the general population. Designing a surveillance program by integrating laboratory data and administrative data could inform PCPs on comorbidities such as diabetes for NAFLD, but validation of the diabetes-related ICD codes in a NAFLD population is required.

To that end, we designed this study with the focus on detecting and reporting diabetes in patients with NAFLD. Our primary objective was to assess the accuracy of diabetes ICD coding in administrative databases among a cohort of confirmed NAFLD patients. Our secondary objectives were to assess inpatient EMR data, visit data, geographical data, and BMI, and to assess how they could be used to peripherally confirm the accuracy of diabetes codes.

## Methods

### Cohort selection: Calgary NAFLD population and data linkage

The Calgary NAFLD Pathway Database (CNPD) was established in 2016 to identify primary care patients with incident NAFLD in the Calgary metropolitan area [10]. NAFLD suspected patients with initial abnormal alanine aminotransferase levels, diabetes mellitus or metabolic syndrome undergo stepped clinical protocols (Additional File 9). Patients’ medications and lifestyle are reviewed by physicians while laboratory tests are initiated to rule out other causes of liver diseases. Only patients formally diagnosed with NAFLD are recorded in the CNPD database. CNPD collects and records demographics and administrative details at the time of shear wave elastography (SWE) testing, and SWE diagnosis information [10]. SWE is a non-invasive imaging technique employed by clinicians to diagnose liver tissue stiffness and identify NAFLD stages [14]. Patients enter CNPD at differing stages of NAFLD based on initial clinician assessment. There were approximately 12,012 patients enrolled in this database at the end of the CNPD study (April 2022). SWE results contained in CNPD were validated and confirmed NAFLD status and stage.

We deterministically linked the CNPD cohort to the following administrative health databases and inpatient EMR using a previously established process [15]: physician claims, NACRS, DAD, pharmaceutical information network (PIN), laboratory database, and Sunrise Clinical Manager (SCM) EMR. Alberta has a unique lifetime identifier known as the Personal Health Number (PHN) which can be used to trace the healthcare utilization of individuals. Inpatient administrative databases have assigned codes which points to EMR encounter records. PHN, dates of visits, and these access codes were used to access and pull required sub-tables. Data from the five-year period prior to SWE and NAFLD diagnosis were linked and extracted from these databases. These databases are under the jurisdiction of Alberta Health and Alberta Health Services. Brief descriptions of these databases are provided below.

1. Physician claims: collects all physician-submitted ICD-9 billing codes from outpatient and inpatient care.
2. DAD: collects all ICD-10-CA codes from inpatient care.
3. NACRS: collects all emergency and outpatient ICD-10-CA codes.
4. PIN: collects all pharmacy dispensed medications details in community settings.
5. Lab: collects all laboratory test results from outpatient and inpatient care.
6. SCM EMR data: inpatient EMR records. Specifically, information tables on intake, discharge, and laboratory data were extracted.

Other data such as visits, geographical data, and BMI were extracted and presented in our work for future comparisons of our cohort to other cohorts in future studies.

### Defining outcome and predictor/feature variables

Our outcome of interest was diabetes coding within the NAFLD population. We defined diabetes categories following the Diabetes Canada Clinical Practice Guidelines [16] by using laboratory hemoglobin A1c [17–20] and supplemented this phenotyping algorithm with diabetes medication data (Additional file 1). It should be noted that different jurisdictions may have different laboratory thresholds for defining diabetes. Specifically, absence of diabetes was defined as the highest HbA1c laboratory result below 6.1% [18, 19] with no evidence of prescribed and fulfilled medications. Prediabetes was defined as HbA1c between 6.1 and 6.4% or an oral glucose tolerance test or random plasma glucose test or fasting plasma glucose test exceeding the thresholds listed in the Diabetes Canada Guidelines [16]. Diabetes category of meeting glycemic target was defined as (a) HbA1c between 6.4 and 7.0%, if no evidence of medication, and (b) HbA1c values < 7.0%, with evidence of prescribed and fulfilled medications. Diabetes category of not meeting glycemic target was defined as the highest HbA1c laboratory result above 7.0% [20]. The presence of fast plasma glucose ≥ 7.0 mmol/L or 2-hour plasma glucose in a 75 g oral glucose tolerance test ≥ 11.1 mmol/L or Random plasma glucose ≥ 11.1 mmol/L gave indication of diabetes in addition to HbA1c values. Intensified therapies such as (a) GLP1RA if obese or having cerebrovascular disease or stroke, and (b) SGLT2 if chronic kidney disease or albuminuria or cerebrovascular disease, were included as a part of the algorithm. This was achieved by applying Quan’s [21] ICD algorithm on cerebrovascular disease, stroke, chronic kidney disease, and cerebrovascular disease to define the sub-cohorts, and then checked for the presence of those medications.

Anatomical therapeutic classification (ATC) and drug identification numbers (DIN) were extracted from PIN to identify diabetes medications. These medication groups included insulin, oral hypoglycemic drugs, biguanides, Glucagon like peptide-1 receptor agonists, dipeptidyl peptidase-4 inhibitors, sulfonylureas, and thiazolidinediones, and sodium-glucose transporter-2 inhibitors (Additional File 1). Dates were checked to precede NAFLD diagnosis date. The list of diabetes medications was developed and assessed by physician authors of this study. Specific categorical variables were created for patients meeting HbA1c values but not receiving medications for later analytical steps. The list was validated against Canada’s drug product database [22] based on the active ingredients and their activity status was confirmed.

The presence of diabetes ICD codes, as defined by Quan et al. [21] utilizing the standard of 2 outpatient physician claims or 1 hospital discharge diagnosis [23], determined whether a patient had diabetes, based on ICD-10 codes E10.0 to 14.7 [21, 23]. We also introduced a basic algorithm requiring either one physician claim or one hospital discharge diagnosis to enhance the verification of our findings. We abstracted from the physician claims database the number of visits to inpatient and outpatient care providers by each patient within five years prior to NAFLD assessment. Five years was considered clinically sound taking into the account the conditions onset [24] which typically takes 3–7 years to fully manifest. This timeframe also allowed for the identification of diabetes using a well-established validation algorithm (2 physician claims or 1 hospitalization within a 2-year period). Geographical data from DAD and physician claims were used to define rural/urban status of patients. Continuous body mass index (BMI) was calculated from weight in kg/height in m^2^ data available from the CNPD database. Hospitalization record details (intake, progress of care, and discharge status) were extracted from SCM EMR which validated records in administrative databases. Sex was coded as male or female. Postal code from physician claims and DAD/NACRS were converted to identify geographic location (urban/rural status). We determined continuous age at the time of registry entry by subtracting the date of birth from the recorded NAFLD confirmation date. Physician claims data contained the type of physician responsible for billing and their practice settings (community, emergency, inpatient, and diagnostic settings). Laboratory data from inpatient EMR was also evaluated and compared against the laboratory database for data completeness. PIN data contained ATC and DIN codes for all fulfilled community dispensed medications. We used Additional file 1 to identify patients who received these drugs and created a variable representing the treatment status.

### Analysis

Descriptive statistics were calculated for the four diabetes cohorts. Demographic and other basic patient characteristics such as age, sex, and Charlson comorbidities were reported. The total numbers of visits with distinct types of physicians were calculated. The DIN codes of the medications listed in Additional file 1 were compared against the PIN database to assess whether patients were being treated for diabetes.

The presence of ICD codes for diabetes was compared against the reference diabetes severity established above. Performance measures, sensitivity, specificity, positive predictive value (PPV), and negative predictive value (NPV) were calculated. We assessed the accuracy of diabetes codes within various categories, including medication (treated), medication (untreated), and two sub-cohorts: oHbA1c between 6.4 and 7.0%, and HbA1c above 7.0%.

The total numbers of true positives, true negatives, false positives, and false negatives were identified for these four diabetes groups. These categories were compared using appropriate statistical techniques, such as the t-test and chi-square test for respective data types. A *p*-value cut-off point of 0.05 was used for bivariate analysis. Non-parametric tests such as the Mann Whitney U test were used in cases where data was not normally distributed. Additionally, we used a time-series analysis to assess for diabetes remission status within each cohort as defined by the Diabetes Canada Clinical Practice Guidelines [25]. These individuals may have had a HbA1C > 6.5% on one occasion but then their HbA1c dropped below this threshold and was maintained there without any antihyperglycemic agents. The latest interpretation closest to the NAFLD diagnosis date per each patient was reported.

The Conjoint Health Research Ethics Board at the University of Calgary approved this study (REB-20-1127). All methods were performed in accordance with the Declaration of Helsinki. Python version 3.1.1 (Python Software Foundation, https://www.python.org/) and R [26] was used for data extraction, cleaning, and parts of the analyses. Appropriate R packages (e.g., rpy2) were imported into Python for statistical analyses.

## Results

### Data linkage

The CNPD database recorded a total of 12,012 patients diagnosed with NAFLD. All patients were linked successfully to the administrative databases. Data linkage to SCM EMR data linked a total of 3,545 patients (29.5%) accounting for 8,425 admissions. These inpatient visits (*n* = 8,425) accounted for an exceedingly small proportion of the total 1.63 million healthcare visits. Table 1 provides the demographics and comorbidities of the patients with and without coded diabetes. Laboratory data retrieved from SCM was matched with inpatient laboratory records from the lab database, achieving a 100% match rate. Extracted information accounted for: 1.6 million records from PIN, 16.6 million records from Physician claims, 9 million records from laboratory data, and a total of 7,268 hospitalization records. This informed us empirically that NAFLD was a dominantly outpatient managed disease. The performance of the standard diabetes algorithm (2 outpatient claims or 1 hospital discharge code) and the minimal code (1 outpatient claim or 1 hospital discharge) prevalence did not differ.

The patients with coded diabetes were older than those with the absence of diabetes codes (mean 57.4 vs. 51.2). Both groups predominately resided in urban areas (92.5 and 93.3%. respectively) which reflects the cohort selection process of the CNPD database. Additionally, individuals with coded diabetes exhibited a higher prevalence of Charlson comorbidities in comparison to those without diabetes codes.

**Table 1:** Demographics and comorbidities in CNPD cohort with coded and non-coded diabetes within five years prior to NAFLD diagnosis

	Diabetes Coded in Administrative Data (*N* = 3,702, %)	Diabetes Not Coded in Administrative Data (*N* = 8,310, %)	*P*-value
**Demographics**			
Age (mean, sd)	57.4 (12.0)	51.2 (13.4)	< 0.01
Male Sex (N, %)	1691 (45.7)	4067(48.9)	< 0.01
BMI (mean, sd)	26.7 (13.9)	25.4 (15.3)	< 0.01
Urban Region (N, %)	3426 (92.5)	7755 (93.3)	0.11
**Charlson Comorbidities** (N, %)			< 0.01
0 and 1	0 (0)	5015 (60.3)	
2	466 (12.6)	2199 (26.5)	
3	1344 (36.3)	816 (9.8)	
4+	1892 (51.1)	1280 (15.4)	

The performance of diabetes ICD codes, as defined by Quan et al. [21], was assessed and are shown in Table 2. Diabetes coding performance showed a sensitivity of 0.81 and a PPV of 0.87. Among patients who met glycemic control, a sensitivity of 0.58 and a PPV of 1.0 was found. The diabetes cohort not meeting glycemic control showed a sensitivity of 0.98 and a PPV of 1.0.

**Table 2:** Performance of diabetes codes against diabetes groups within five years to NAFLD diagnoses

		Performance Indicators				
	Ratio of having diabetes related ICD codes (%), N	Sensitivity	Specificity	PPV	NPV	Accuracy
NAFLD (*n* = 12,012)	32.3	0.81	0.94	0.87	0.91	0.90
Absence of Diabetes (*n* = 7,112)	5	NA	0.95	NA	1.0	0.95
Prediabetes (*n* = 889)	17	NA	0.83	NA	1.0	0.83
Meeting glycemic Targets (*n* = 1,744)	58	0.58	NA	1.0	NA	0.58
Diabetes – Not meeting glycemic Diabetes (*n* = 2,267)	98	0.98	NA	1.0	NA	0.98

### Error rates within severity sub-cohorts

Among those with the absence of diabetes, 6,789 were true negative cases and 323 were false positive cases, representing a diabetes coding error rate of 4.5% over the 5-year period. Patients with HbA1c values above 7.0% had a total of 31 false negative cases and 1426 true positive cases, representing an error rate of 2.2% in the same period. The diabetic meeting glycemic control group (HbA1c between 6.4 and 7.0%) had a total of 736 false negatives and 1,008 true positives, resulting in a 42.2% coding error rate. Upon further investigations it was discovered that a total of 536 among 736 false negatives had received diabetic medications and met glycemic targets.

Tables 3 and 4 presents a comparison of comorbidities and healthcare utilization among patients who achieved glycemic targets (HbA1c group of between 6.4 and 7.0%). Specific comparisons for this HbA1c subgroup are shown in supplementary materials (Additional files 2 to 7). Notably, the number of emergency GP visits were statistically significant and ambulatory specialist visits approached the *p*-value threshold of 0.05 (*p* = 0.07). Slightly different visitation patterns were observed in the HbA1c greater than 7.0% groups. Among those with HbA1c greater than 7.0%, the false negative groups had fewer visits to community GPs (mean 57.5 vs. 70.8), and fewer to community specialists (mean 31.5 vs. 59.6) then true positives cases. Additional File 8 provides a detailed description of the diabetic remissions status, as outlined in the methods section. (Additional File 8).

**Table 3:** Demographic and comorbidities among patients with true positive and false negative among patients meeting glycemic targets (HbA1c group between 6.4 and 7.0%)

	Diabetes, meeting glycemic targets		
	True Positive (*N* = 1,008)	False Negative (*N* = 736)	*P*-value
**Demographics**			
Age (mean, sd)	56.2 (12.7)	51.1 (13.2)	0.40
Male Sex (N, %)	429 (42.6)	251 (34.1)	0.69
BMI (mean, sd)	34.5 (9.8)	35.8 (10.2)	0.80
Region (Urban, %)	945 (94.8)	701 (95.5)	0.92
**Charlson Comorbidities** (N, %)			< 0.01
0 and 1	0 (0.0)	412 (55.6)	
2	128 (12.7)	187 (25.4)	
3	381 (37.8)	95 (12.9)	
4+	499 (49.5)	52(6.1)	

**Table 4:** Comparisons of number of healthcare providers seen among true positives, and false negatives among patients meeting glycemic targets (HbA1c group between 6.4 and 7.0%)

	Diabetes, meeting glycemic targets		
	True Positive (*N* = 1,008)	False Negative (*N* = 736)	*P*-value
**Community**			
GP Mean (Median, IQR)	65.1 (50.5, 43.0)	69.1 (50.0, 43.0)	0.99
Specialist Mean (Median, IQR)	49.2 (29.0, 42.0)	47.7 (26.0, 38.0)	0.16
Allied Mean (Median, IQR)	7.0 (3.0, 6.0)	4.7 (2.0, 5.0)	< 0.01
**Emergency**			
GP Mean (Median, IQR)	1.3 (0.0, 0.0)	1.4 (0.0, 0.0)	0.04
Specialist Mean (Median, IQR)	4.2 (2.0, 4.0)	5.1 (2.0, 4.0)	0.52
Allied Mean (Median, IQR)	0.001 (0.0, 0.0)	NA	NA
**Inpatient**			
GP Mean (Median, IQR)	1.7 (0.0, 0.0)	2.4 (0.0, 0.0)	0.59
Specialist Mean (Median, IQR)	8.0 (1.5, 4.0)	10.5 (2.0, 6.0)	0.50
Allied Mean (Median, IQR)	NA	0.007 (0.0, 0.0)	0.11
**Diagnostic Therapy**			
GP Mean (Median, IQR)	0.008 (0.0, 0.0)	0.02 (0.0, 0.0)	0.47
Specialist Mean (Median, IQR)	23.1 (15.0, 17.0)	23.5 (14.0, 16.0)	0.27
Allied Mean (Median, IQR)	0.4 (0.0, 2.0)	0.3 (0.0, 0.0)	0.35
**Ambulatory (Other)**			
GP Mean (Median, IQR)	0.04 (0.0,	0.02 (0.0, 0.0)	0.30
Specialist Mean (Median, IQR)	2.1 (0.0,	2.2 (0.0, 1.0)	0.07

*Presented *p*-values are based on post-hoc tests with Bonferroni corrections applied on Kruskal-Wallis ANOVA test. Allied health represents allied health providers and professionals. Allied health providers cover oral and maxillofacial surgery, optometry, laboratory medicine, diagnostic imaging, podiatry, and podiatric surgery, while allied health professionals cover the areas of pain, speech, and other physical challenges

The remission status on diabetes closest to the NAFLD diagnosis date is reported in Additional File 8 and indicates that most individuals remained in their diabetic categories at the time of NAFLD diagnosis.

## Discussion

In this study, we examined the accuracy of diabetes severity coding in the NAFLD population by linking the NAFLD registry to multiple administrative and EMR databases. The primary aim was to identify predictive factors associated with error within the diabetes cohorts. In this study cohort it was observed that diabetes coding accuracy was not dependent on whether a patient received treatment with community-dispensed medications. The coding error among patients with clear indications of diabetes (HbA1c greater than 7.0%) was 6.4% (31/1,426), whereas among those without diabetes (HbA1c less than 6.1%) the error rate was 4.5% (323/7112) over a five-year period. In contrast, not meeting glycemic control group exhibited a considerably higher coding error rate of 42.2%.

In Canadian health systems, primary care physicians may submit up to three ICD codes as part of physician billing, which are compiled into claims databases [27]. Furthermore, physicians are only required to submit one code representing the commonly completed diagnoses during the patient encounter. Nearly all physician visits related to diabetes during the 5-year period took place in primary care settings, accounting for 99.9% of visits (1.629 million out of 1.630 million visits). However, it is noteworthy that 45.9% of the study cohort experienced at least one inpatient admission. Consequently, approximately half of the cohort had other primary medical conditions that were being managed and diabetes might have been considered as a comorbidity. It is hypothesized that the underreporting of diabetes codes in the glycemic control group may be due to this factor, contributing to the observed coding inaccuracies. The identification of 536 out of 736 false negatives within the cohort meeting glycemic control criteria, who also had documented prescriptions for diabetes medications and maintained a HbA1c control, further supports this observation. Despite linking to impatient EMRs and other administrative data in the 5-year period leading up to the NAFLD diagnosis, no specific details for the rationale for coding were obtained.

The list of ICD codes originally developed for defining diabetes was for identifying comorbidities for calculating the risk of mortality as part of the Charlson algorithm [1] undergone multiple revisions [21, 28]. These refined ICD code-based algorithms are used for syndromic public health surveillance of chronic conditions [3, 4, 29]. Primarily, diabetes codes are employed in prevalence studies to determine the presence of the disease within the population [30–33]. These prevalence studies play a pivotal role in informing health systems and guiding the planning for control strategies. Therefore, understanding coding errors is essential for evaluating and refining existing health programs and keeping health databases up to date.

It is worth noting that, from our current understanding, diabetes cohorts have not been adequately considered in existing literature when assessing ICD code accuracy. This study indicated that the cohorts at the extremes (i.e., the highest and lowest A1c groups) demonstrated relatively precise ICD coding accuracy. However, the diabetes cohort in the glycemic control group encountered challenges, likely stemming from the structure of the ICD code submission system and, possibly a lack of coding, as diabetes is often presumed to be a well-managed comorbidity. To address these issues, we propose a few solutions to mitigate this for the boundary group. Currently, the DAD allows the submission of up to twenty-five diagnostic codes for acute facility admission encounters, regardless of payment status [34]. Expanding the scope of physician claims beyond three codes may provide a more comprehensive understanding of patient profiles. However, this may not be easy to achieve given the complexities and barriers involved in processes for creating administrative data [35, 36]. Linkage to inpatient EMR confirmed the quality of extracted laboratory data and provided limited clinical context associated with lack of diabetes coding justification in this patient cohort. Connecting existing data systems with primary care EMRs and directly phenotyping diabetes from clinical notes may offer additional clinical context and contribute to enhancing the accuracy of ICD codes collected within administrative databases.

This study has several limitations. First, the claims database uses ICD-9 codes while DAD and NACRS use ICD-10 codes, and coding standards between the two could be different. Second, obtaining a comprehensive clinical context behind coding rationale can be challenging, as detailed data on patients, providers and context may not always be available. Third, our reference standard may not be perfect and there is a possibility that some diabetes cases were not phenotyped properly. Nevertheless, we followed clinical care practice guideline, and our observations align with clinical expectations. Lastly, the clinical and administrative data utilized in this study are specific to one city in a single Canadian province, and thus may not be generalizable to other settings. Additional external validation in diverse contexts is warranted.

Despite these limitations, this study offers a detailed assessment on coding accuracy for diabetes severity groups. Similar analyses could be conducted on other chronic conditions, contributing to the improvement of chronic disease surveillance programs. Furthermore, there is potential to enhance surveillance through ongoing research activities, including the incorporation of patient-reported outcomes and the artificial intelligence. The integration of self-reported diabetes data from patients [37, 38] into existing health system infrastructure, coupled with the development and deployment of self-reported tools via recommender systems [39], can complement the quality of administrative databases and address these limitations.

## Conclusions

In summary, ICD codes demonstrate strong performance in identifying individuals without diabetes and those who do not meet glycemic control within the NAFLD population. However, the codes did not perform well for accurately identifying diabetes cases meeting glycemic control. Patients with false negative diabetes-related ICD-codes often exhibit evidence of glycemic control and receiving medications, highlighting the need for a more comprehensive clinical context, which may require additional data linkage from primary care settings. Our study provides insights on accuracy of diabetes coding among NAFLD population, and similar methodologies can be employed on to assess other chronic conditions.

### Electronic supplementary material

Below is the link to the electronic supplementary material.

Supplementary Material 1

## Data Availability

The data that support the findings of this study are available from Alberta Health Services and Alberta Health, but restrictions apply to the availability of these data, which were used under license for the current study, and so are not publicly available. Data are however available from the authors upon reasonable request and with permission of Alberta Health Services and Alberta Health.

## Abbreviations

ATC: Anatomical therapeutic classification
CNPD: Calgary NAFLD pathway database
DAD: Discharge abstract database
DIN: Drug identification number
EMR: Electronic Medical Records
GP: General practitioner
HbA1c: Hemoglobin A1c
ICD: International Classification of Diseases
NACRS: National ambulatory care reporting system
NAFLD: Non-alcoholic fatty liver disease
NASH: Non-alcoholic steatohepatitis
NPV: Negative predictive value
PCP: Primary care providers
PIN: Pharmaceutical information network
PPV: Positive predictive value
SCM: Allscripts sunrise clinical manager
SWE: Shear wave elastography
