# Supplementary material for: Evaluating the coding accuracy of type 2 diabetes mellitus among patients with non-alcoholic fatty liver disease
Source: BMC Health Serv Res. 2024 Feb 16;24:218. doi: 10.1186/s12913-024-10634-8 (PMC10874028; doi:10.1186/s12913-024-10634-8)

**Updated BMC Health Services Diabetes Tables**

**Defining Categories based on HbA1c and medications (following Diabetes Canada Clinical Practices)**

1. Absence of diabetes: Highest HbA1c laboratory result below 6.1%, with no evidence of prescribed and fulfilled medications.
2. Pre-diabetes: defined by A1C 6.1-6.4% or OGTT or RPG or FPG using the thresholds in the Diabetes Canada guidelines.
3. Meeting glycemic targets:
   1. HbA1c between 6.4 and 7.0%, if no evidence of medication
   2. HbA1c values < 7.0%, with evidence of prescribed and fulfilled medications.
      1. HbA1c values can drop to much lower ranges with medications according to literature, a lower limit is not set
4. Diabetes (Not meeting glycemic targets): HbA1c >7% (
   - On appropriately intensified therapy:
     1. GLP1RA if obese or CVD or stroke
     2. SGLT2 if CKD or albuminuria or CVD
   - Also not receiving indicated pharmacotherapy.

**Additional File 1**. List of diabetes medication, types, and DIN

| **Diabetes Drug Type** | **Drug Identification Number (DIN)** |
| --- | --- |
| Short-acting insulin | Regular insulin (Humulin and Novolin)  01962639, 00889113, 01962655, 00889105, 00795879, 01959212, 01962647, 00889091, 01962663, 00889121, 00587737, 01959239, 02403447, 02241310, 00586714, 02415089, 01959220, 00733075 |
| Rapid-acting insulins | Insulin aspart  02460408, 02460416, 02460424, 02520974, 02520982, 02265435, 02244353, 02245397, 02377209 |
|  | Insulin glulisine  02279460, 02279479, 02279487, 02294346 |
|  | Insulin lispro (Humalog)  02229704, 02470152, 02229705, 02403412, 02439611, 02240294, 02403420, 02240295, 02240297, 02403439, 02241283, 02469898, 02469901, 02469871, 02506564, 02506572, 02529254 |
| **Intermediate-acting insulin** | Insulin isophane (Humulin N, Novolin N)  01962639, 00889113, 01962655, 00889105, 00795879, 01959212, 01962647, 00889091, 01962663, 00889121, 01959239, 02403447, 02024306, 02024217, 02025248, 02024314, 02024322, 02024225, 02024268, 02024446, 02024403, 02024322, 02024225, 02024268, 02024233, 02024284, 02024314 |
| **Long-acting insulins** | Insulin degludec  02467860, 02467879, 02467887, 02474875, 02474875 |
|  | Insulin detemir  02412829, 02271842 |
|  | Insulin glargine  02444844, 02461528, 02245689, 02251930, 02294338, 02526441, 02478293, 02478293, 02493373, 02441829 |
|  |  |
| **Combination insulins** | Humalog Mix 75/25 (insulin lispro protamine-insulin lispro)  02240294, 02403420, 02240295 |
|  | Humalog Mix 50/50 (insulin lispro protamine-insulin lispro)  02240297, 02403439 |
|  | Humulin 70/30 (human insulin NPH-human insulin regular)  00795879, 01959212 |
|  | Novolin 70/30  02024217, 02025248 |
| Biguanides | Metformin (Glucophage, Metformin Hydrochloride ER, Glumetza, Riomet, Fortamet)  02099233, 02162849, 02446065, 02162822, 02229517, 02231389, 02238827, 02242793, 02242794, 02246965, 02284782, 02284790, 02343606, 02343614, 02353377, 02353385, 02378841, 02378868, 02385341, 02385368, 02268493, 02300451, 02268507  Metformin-alogliptin (Kazano)  02417219, 02417227, 02417235 |
|  | Metformin-canagliflozin (Invokamet)  02455404, 02455412, 02455420, 02455439, 02455447, 02455455, 02477394, 02477408, 02477416, 02477424 |
|  | Metformin-dapagliflozin (Xigduo XR)  02449935, 02449943 |
|  | Metformin-empagliflozin (Synjardy)  02456575, 02456583, 02456591, 02456605, 02456613, 02456621 |
|  | Metformin-linagliptin (Jentadueto)  02403250, 02403269, 02403277 |
|  | Metformin-rosiglitazone (Avandamet)  02247085, 02247086, 02247087, 02248440, 02248441 |
|  | Metformin-saxagliptin (Kombiglyze XR)  02389169, 02389177, 02389185 |
|  | Metformin-sitagliptin (Janumet)  02333864, 02333872, 02416786, 02416794, 02416808 |
| Dipeptidyl peptidase-4 (DPP-4) inhibitors | Alogliptin (Nesina)  02417189, 02417197, 02417200 |
|  | Alogliptin-metformin (Kazano)  02417219, 02417227, 02417235 |
|  | Alogliptin-pioglitazone (Oseni)  02419300, 02419319, 02419327, 02419335, 02419343, 02419351 |
|  | Linagliptin (Tradjenta)  02370921 |
|  | Linagliptin-empagliflozin (Glyxambi)  02459752, 02459760 |
|  | Linagliptin-metformin (Jentadueto)  02403250, 02403269, 02403277 |
|  | Saxagliptin (Onglyza)  02333554, 02375842 |
|  | Sexagliptin (Apo-Sexagliptin)  02507471, 02507498, |
|  | Sexagliptin (Sandoz-Sexagliptin)  02468603, 02468611 |
|  | Saxagliptin-metformin (Kombiglyze XR)  02389169, 02389177, 02389185 |
|  | Sitagliptin (Januvia)  02303922, 02388839, 02388847 |
|  | Sitagliptin-metformin (Janumet and Janumet XR)  02333856, 02333864, 02333872, 02416786, 02416794, 02416808 |
| Glucagon-like peptide-1 receptor agonists (GLP-1 receptor agonists) | Dulaglutide (Trulicity)  02448572, 02448580, 02448599, 02448602, 02530163, 02530171 |
|  | Exenatide (Byetta)  02361809, 02361817 |
|  | Exenatide extended release (Bydureon)  02448610, 02483203 |
|  | Liraglutide  02437899, 02351064, 02474875 |
|  | Semaglutide  02471469, 02471477, , 02523930, 02497581, 02497603, 02497611, 02522551, 02522578, 02522586, 02522594, 02522608, 02528509, 02528517, 02528525, 02528533, 02528541 |
| GLP-1 insulin combinations | (Xultophy, Soliqua) 02474875, 02478293 |
| Meglitinides | Nateglinide (Starlix)  02245439, 02245440, 02245438 |
|  | Repaglinide  02239926, 02239925, 02239924, 02355663, 02355671, 02355698, 02424258, 02424266, 02424274, 02321475, 02321483, 02321491, 02354926, 02354934, 02354942, 02357453, 02357461, 02357488 |
| Alpha-glucosidase inhibitor | Acarbose  02493780, 02493799, 02190885, 02190893, 02494078, 02494086 |
| Sodium-glucose transporter (SGLT) 2 inhibitors | Dapagliflozin (Farxiga)  02435462, 02435470 |
|  | Dapagliflozin-metformin (Xigduo XR)  02449935, 02449943 |
|  | Canagliflozin (Invokana)  02425483, 02425491 |
|  | Canagliflozin-metformin (Invokamet)  02455404, 02455412, 02455420, 02455439, 02455447, 02455455, 02477394, 02477408, 02477416, 02477424 |
|  | Empagliflozin (Jardiance)  02443937, 02443945 |
|  | Empagliflozin-linagliptin (Glyxambi)  02459752, 02459760 |
|  | Empagliflozin-metformin (Synjardy)  02456575, 02456583, 02456591, 02456605, 02456613, 02456621 |
|  | Ertugliflozin (Steglatro) empagliflozin-metformin (Synjardy)  02456575, 02456583, 02456591 |
| Sulfonylureas | Glimepiride  02245272, 02245273, 02245274, 02269589, 02269597, 02269619 |
|  | Glimepiride-rosiglitazone (Avandaryl)  02258781, 02258803, 02258811 |
|  | Gliclazide  02483300, 02483319, 02245247, 02297795, 02407124, 02363518, 00765996, 02242987, 02356422, 02248210, 02287072, 02155850, 02248453, 02429764, 02429772, 02423286, 02423294, 02229519, 02438658, 02449765, 02336316, 02294400, 02254719, 02461323, 02461331, 02439328, 02463571, 02238103 |
|  | Glyburide  01913654, 01913662, 02234514, 00720941, 01959352 02350459, 02350467, 02485664, 02236734, 01913670, 01913689 02224550, 02224569, 00012599, 00454753, 01987836, 01987534 |
|  | Chlorpropamide  00399302, 00312711, 00024708, 00024716 |
|  | Tolbutamide  00013889, 00021849, 00012602, 00012610, 00312762, 00156663, 00431168 |
| Thiazolidinediones | Rosiglitazone  02403366, 02403374, 02403382, 02241112, 02241113, 02241114 |
|  | Rosiglitazone-glimepiride (Avandaryl)  02258781, 02258803, 02258811 |
|  | Pioglitazone  02339587, 02339595, 02391600 , 02302861, 02302888, 02302896 , 02374587, 02374595, 02302942, 02302950 , 02302977, 02365529, 02365537, 02397307, 02326477, 02326485, 02326493, 02303124, 02303132, 02303140, 02389290, 02389304, 02389312, 02242572, 02242573, 02242574 |
|  | Pioglitazone-alogliptin (Oseni)  02419300, 02419319, 02419327, 02419335, 02419343, 02419351 |

**Group 1: Absence of Diabetes**

**Additional File 2.** Comparisons of demographics and comorbidities among true negatives and false positives within absence of diabetes group.

|  | Absence of Diabetes | |  |
| --- | --- | --- | --- |
|  | True Negative    (N= 6,789) | False Positive    (N= 323) | P-value |
| **Demographics** |  |  |  |
| Age (mean, sd) | 50.5 (13.4) | 56.4 (13.1) | 0.09 |
| Male Sex (N, %) | 3,464 (51.0) | 146 (45.2) | 0.06 |
|  |  |  |  |
| BMI (mean, sd) | 31.9 (9.9) | 32.5 (8.4) | 0.30 |
| Region (Urban, %) | 6,319 (93.1) | 303 (93.8) | 0.33 |
|  |  |  |  |
| **Charlson Comorbidities** (N, %) |  |  | <0.01 |
| 0 and 1 | 4,168 (61.4) | 0.0 (0.0) |  |
| 2 | 1,722 (25.4) | 30 (9.3) |  |
| 3 | 626 (9.2) | 126 (39.0) |  |
| 4+ | 273 (4.0) | 167 (51.7) |  |

**Additional File 3.** Comparisons of total number of healthcare providers seen among true negatives and false positives within absence of diabetes group.

|  | Prediabetes | |  |
| --- | --- | --- | --- |
|  | True Negative  (N= 6,789) | False Positive  (N= 323) | P-value |
| **Community** |  |  |  |
| GP Mean  (Median, IQR) | 46.7 (36.0, 32.0) | 61.2 (49.0, 34.5) | <0.01 |
| Specialist Mean  (Median, IQR) | 31.8 (17.0, 26.0) | 42.3 (25.0, 34.5) | <0.01 |
| Allied Mean  (Median, IQR) | 3.8 (1.0, 4.0) | 5.8 (3.0, 5.0) | <0.01 |
|  |  |  |  |
| **Emergency** |  |  |  |
| GP Mean  (Median, IQR) | 1.2 (0.0, 0.0) | 2.0 (0.0, 0.0) | <0.01 |
| Specialist Mean  (Median, IQR) | 3.1 (0.0, 3.0) | 4.5 (2.0, 4.0) | <0.01 |
| Allied Mean  (Median, IQR) | 0.001 (0.0, 0.0) | NA |  |
|  |  |  |  |
| **Inpatient** |  |  |  |
| GP Mean  (Median, IQR) | 1.2 (0.0, 0.0) | 2.1 (0.0, 0.0) | <0.01 |
| Specialist Mean  (Median, IQR) | 5.8 (0.0, 3.0) | 7.1 (2.0, 6.0) | <0.01 |
| Allied Mean  (Median, IQR) | 0.01 (0.0, 0.0) | 0.07 (0.0, 0.0) | <0.01 |
|  |  |  |  |
| **Diagnostic Therapy** |  |  |  |
| GP Mean  (Median, IQR) | 0.008 (0.0, 0.0) | 0.006 (0.0, 0.0) | 0.34 |
| Specialist Mean  (Median, IQR) | 16.5 (11.0, 13.0) | 19.1 (14.0, 15.0) | <0.01 |
| Allied Mean  (Median, IQR) | 0.19 (0.0, 0.0) | 0.2 (0.0, 0.0) | 0.07 |
|  |  |  |  |
| **Ambulatory (Other)** |  |  |  |
| GP Mean  (Median, IQR) | 0.01 (0.0, 0.0) | 0.03 (0.0, 0.0) | 0.63 |
| Specialist Mean  (Median, IQR) | 1.0 (0.0, 0.0) | 1.3 (0.0, 2.0) | <0.01 |

**Group 2: Pre-diabetes**

**Additional File 4.** Comparisons of demographics and comorbidities among true negatives and false positives within pre-diabetes group.

|  | Prediabetes group | |  |
| --- | --- | --- | --- |
|  | True Negative    (N=739) | False Positive    (N=150) | P-value |
| **Demographics** |  |  |  |
| Age (mean, sd) | 57.0 (12.4) | 57.1 (11.0) | 0.58 |
| Male Sex (N, %) | 329 (44.5) | 67 (44.7) | 0.79 |
|  |  |  |  |
| BMI (mean, sd) | 32.4 (6.7) | 33.3 (10.8) | 0.97 |
| Region (Urban, %) | 693 (93.8) | 141 (94.0) | 0.37 |
|  |  |  |  |
| **Charlson Comorbidities** (N, %) |  |  |  |
| 0 and 1 | 407 (55.1) | 0 (0.0) | <0.01 |
| 2 | 196 (26.5) | 14 (9.3) |  |
| 3 | 92 (12.4) | 61 (40.7) |  |
| 4+ | 44 (5.9) | 75 (50.0) |  |

**Additional File 5.** Comparisons of total number of healthcare providers seen among true negatives and false positives within pre-diabetes group.

|  | Prediabetes | |  |
| --- | --- | --- | --- |
|  | True Negative  (N= 739) | False Positive  (N=150) | P-value |
| **Community** |  |  |  |
| GP Mean  (Median, IQR) | 54.9 (41.0, 34.0) | 68.8 (47.0, 33.0) | 0.03 |
| Specialist Mean  (Median, IQR) | 41.0 (24.0, 37.8) | 45.7 (24.0, 44.0) | 0.35 |
| Allied Mean  (Median, IQR) | 5.3 (3.0, 5.0) | 5.8 (3.0, 6.0) | 0.10 |
|  |  |  |  |
| **Emergency** |  |  |  |
| GP Mean  (Median, IQR) | 1.2 (0.0, 0.0) | 1.0 (0.0, 0.0) | 0.91 |
| Specialist Mean  (Median, IQR) | 3.5 (1.0, 3.0) | 3.2 (1.0, 4.0) | 0.90 |
| Allied Mean  (Median, IQR) | NA | NA | NA |
|  |  |  |  |
| **Inpatient** |  |  |  |
| GP Mean  (Median, IQR) | 1.8 (0.0, 0.0) | 0.8 (0.0, 0.0) | 0.60 |
| Specialist Mean  (Median, IQR) | 6.1 (0.0, 3.0) | 6.9 (1.0, 4.0) | 0.61 |
| Allied Mean  (Median, IQR) | NA | NA | NA |
|  |  |  |  |
| **Diagnostic Therapy** |  |  |  |
| GP Mean  (Median, IQR) | 0.01 (0.0, 0.0) | 0.007 (0.0, 0,0) | 0.66 |
| Specialist Mean  (Median, IQR) | 20.1 (14.0, 14.0) | 28.8 (17.0, 18.5) | 0.02 |
| Allied Mean  (Median, IQR) | 0.3 (0.0, 0.0) | 0.2 (0.0, 0.0) | 0.87 |
|  |  |  |  |
| **Ambulatory (Other)** |  |  |  |
| GP Mean  (Median, IQR) | 0.008 (0.0, 0.0) | NA | NA |
| Specialist Mean  (Median, IQR) | 1.3 (0.0, 1.0) | 1.4 (0.0, 2.0) | 0.06 |

**Group 4: Diabetes - glycemic target not met**

**Additional File 6.** Comparisons of demographics and comorbidities among false negatives and true positives within diabetes – glycemic control not met.

FN vs. TP

|  | Diabetes - glycemic control not met | |  |
| --- | --- | --- | --- |
|  | False Negative  (N= 46) | True Positive    (N=2221) | P-value |
| **Demographics** |  |  |  |
| Age (mean, sd) | 53.1 (13.2) | 58.1 (11.6) | 0.01 |
| Male Sex (N, %) | 23 (50.0) | 1049 (47.2) | 0.72 |
|  |  |  |  |
| BMI (mean, sd) | 34.6 (5.5) | 34.2 (8.3) | 0.11 |
| Region (Urban, %) | 42 (91.3) | 2037 (91.7) | 0.92 |
|  |  |  |  |
| **Charlson Comorbidities** (N, %) |  |  | <0.01 |
| 0 and 1 | 28 (60.9) | 0 (0.0) |  |
| 2 | 14 (30.4) | 294 (13.2) |  |
| 3 | 3 (8.3) | 776 (34.9) |  |
| 4+ | 1 (2.2) | 1151 (51.8) |  |

**Additional File 7.** Comparisons of number of healthcare providers seen among false negatives and true positives within uncontrolled diabetes.

|  | Diabetes - glycemic control not met | |  |
| --- | --- | --- | --- |
|  | False Negative  (N= 31) | True Positive  (N=1426) | P-value |
| **Community** |  |  |  |
| GP Mean  (Median, IQR) | 57.5 (40.0, 30.0) | 70.8 (55.0, 41.0) | <0.01 |
| Specialist Mean  (Median, IQR) | 31.5 (21.5, 42.8) | 59.6 (38.0, 54.0) | <0.01 |
| Allied Mean  (Median, IQR) | 3.5 (2.0, 5.0) | 8.2 (5.0, 8.0) | <0.01 |
|  |  |  |  |
| **Emergency** |  |  |  |
| GP Mean  (Median, IQR) | 1.7 (0.0, 0.0) | 1.9 (0.0, 0.0) | 0.86 |
| Specialist Mean  (Median, IQR) | 3.3 (1.0, 2.0) | 5.3 (2.0, 4.0) | 0.32 |
| Allied Mean  (Median, IQR) | NA | 0.001 (0.0, 0.0) | NA |
|  |  |  |  |
| **Inpatient** |  |  |  |
| GP Mean  (Median, IQR) | 1.7 (0.0, 1.0) | 2.8 (0.0, 0.0) | 0.36 |
| Specialist Mean  (Median, IQR) | 3.3 (2.0, 4.0) | 11.6 (2.0, 5.0) | 0.77 |
| Allied Mean  (Median, IQR) | NA | 0.004 (0.0, 0.0) | NA |
|  |  |  |  |
| **Diagnostic Therapy** |  |  |  |
| GP Mean  (Median, IQR) | NA | 0.004 (0.0, 0.0) | NA |
| Specialist Mean  (Median, IQR) | 22.8 (11.5, 11.0) | 22.4 (14.0, 16.5) | 0.31 |
| Allied Mean  (Median, IQR) | 0.1 (0.0, 0.0) | 0.3 (0.0, 0.0) | 0.89 |
|  |  |  |  |
| **Ambulatory (Other)** |  |  |  |
| GP Mean  (Median, IQR) | 0.07 (0.0, 0.0) | 0.03 (0.0, 0.0) | 0.34 |
| Specialist Mean  (Median, IQR) | 1.4 (0.0, 2.0) | 1.6 (0.0, 2.0) | 0.37 |

**Additional File 8.** Determination of Diabetes Remission Status Among Subcohorts.

| **Subcohort** | **Diabetes Status** | **Total Number** |
| --- | --- | --- |
| Absence of diabetes | Remission not detected | 6,985 |
|  | No information available | 127 |
|  |  |  |
| Prediabetes | Remission not yet detected | 868 |
|  | No information available | 21 |
|  |  |  |
| Diabetes – meeting glycemic control | Remission not detected | 1,698 |
|  | Type 2 diabetes relapse | 3 |
|  | Remission to prediabetes | 2 |
|  | Remission to normal glucose levels | 0 |
|  | No information available | 41 |
|  |  |  |
| Diabetes – not meeting glycemic control | Remission not detected | 2217 |
|  | Type 2 diabetes relapse | 1 |
|  | Remission to normal glucose levels | 1 |
|  | No information available | 48 |

**Additional File 9.** NAFLD Primary Care Pathway Flow and Physician Checklist in Calgary.


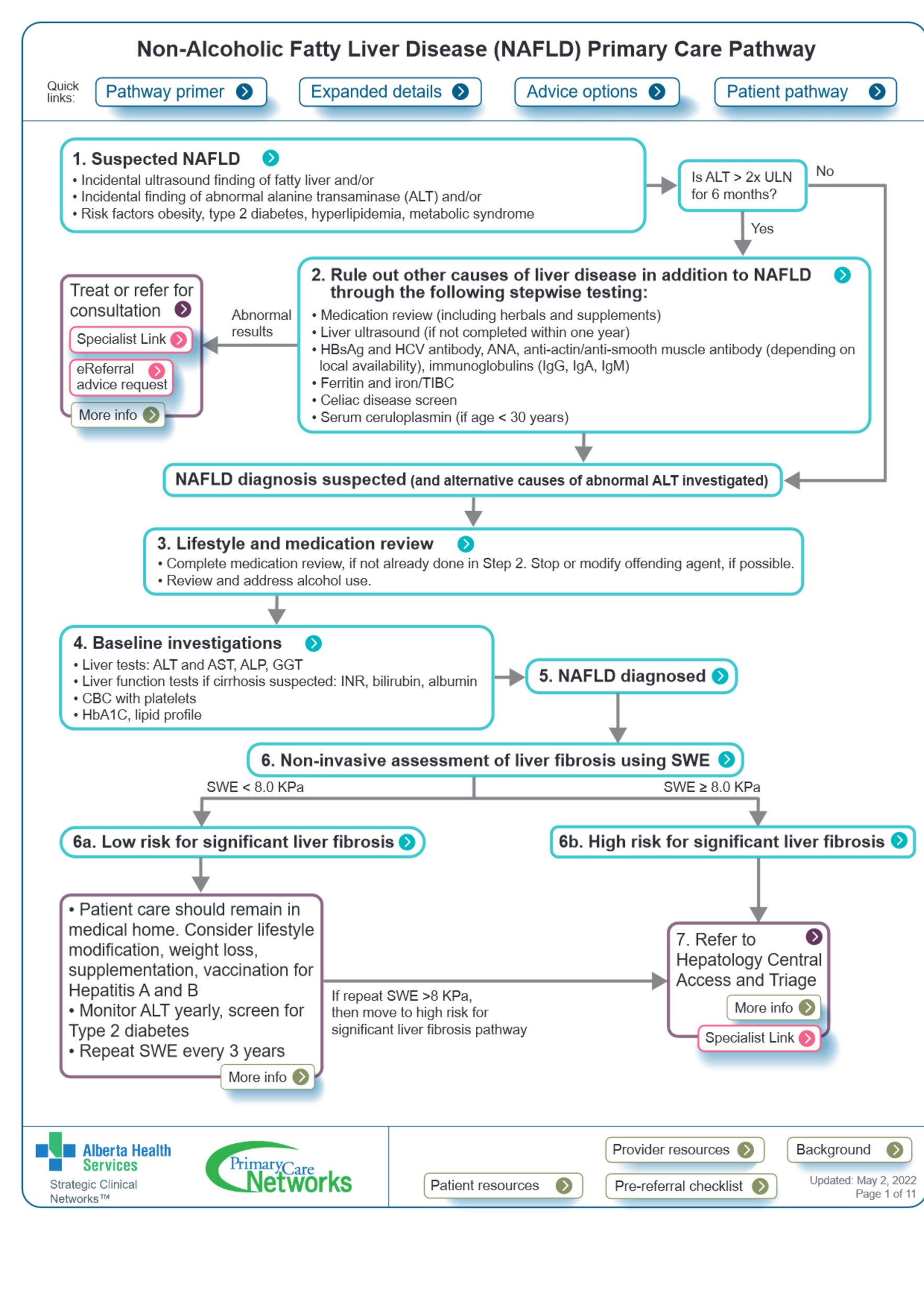


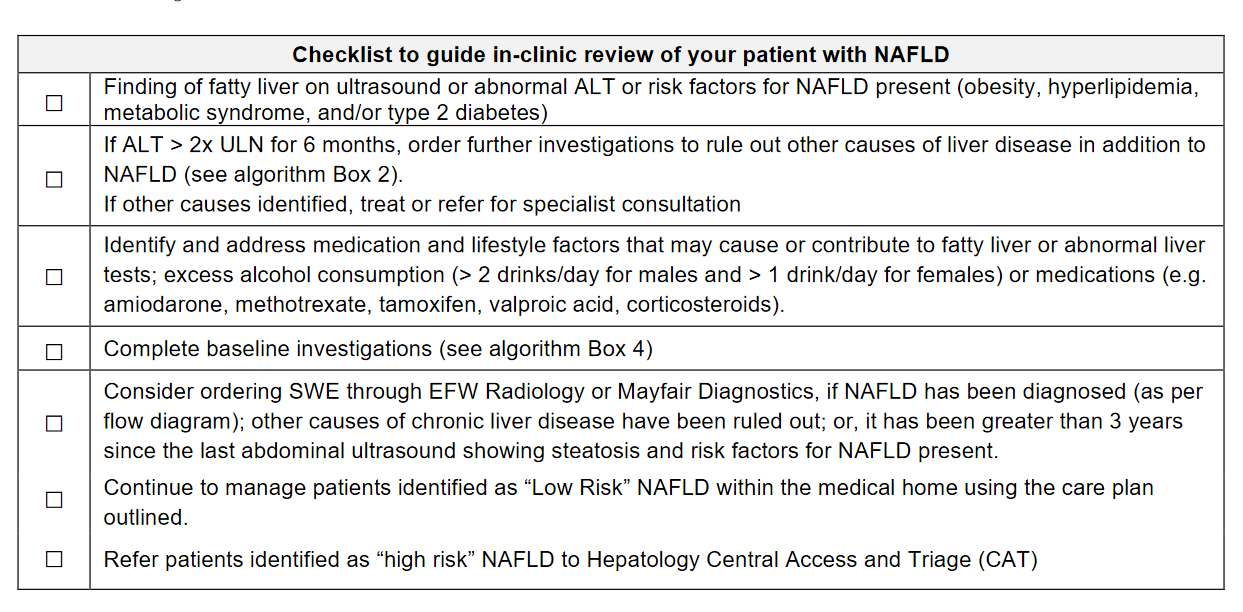

Supplement: Supplementary file 1 — Supplementary Material 1 [file 12913_2024_10634_MOESM1_ESM.docx]
